# Measuring national mood with music: using machine learning to construct a measure of national valence from audio data

**DOI:** 10.3758/s13428-021-01747-7

**Published:** 2022-02-25

**Authors:** Emmanouil Benetos, Alessandro Ragano, Daniel Sgroi, Anthony Tuckwell

**Affiliations:** 1 Queen Mary University of London, London, England UK; 2 The Alan Turing Institute, London, England UK; 3 University College Dublin, Dublin, Ireland; 4 University of Warwick, Coventry, England UK

**Keywords:** Valence, Life satisfaction, Subjective well-being, Music, Machine learning, Data science

## Abstract

We propose a new measure of national valence based on the emotional content of a country’s most popular songs. We first trained a machine learning model using 191 different audio features embedded within music and use this model to construct a long-run valence index for the UK. This index correlates strongly and significantly with survey-based life satisfaction and outperforms an equivalent text-based measure. Our methods have the potential to be applied widely and to provide a solution to the severe lack of historical time-series data on psychological well-being.

**Supplementary Information:**

The online version contains supplementary material available at 10.3758/s13428-021-01747-7.

## Introduction

One of the most fundamental human concerns, life satisfaction, has also become an important issue for behavioral scientists and policymakers at least since 2011 when the O.E.C.D. launched their “Better Life index” and the United Nations released the first edition of the now annual World Happiness Report. The most popular method of data collection has been through national-level surveys of subjective well-being. Recently, in response to the complete absence of any consistent time-series data stretching back more than 50 years, a new measure was developed which utilized the mood embedded within words in books which is often referred to as “text valence” (Hills et al. , 2019). This provides a way to construct life satisfaction data from the period before the availability of survey data but suffers from a number of problems including changes in the meanings of words and the paucity of language data for many developing nations.

Like language, music can also encode emotional information: it has been described as a “language of the emotions” (Cooke , 1959), with studies demonstrating that different people can recognize the same patterns of emotion in a song (Juslin , 2013). Moreover, it is the emotional experience that music offers that primarily motivates individuals to listen to music in the first place (Juslin and Laukka , 2004). Music also has the potential to be a good between-country predictor since it is not only an emotional language but has been called a “universal” language (Longfellow , 1835) and is found in every society (Mehr et al. , 2019). This paper demonstrates music’s potential by showing that the positive mood (valence) embedded within audio data, in particular in a country’s most popular songs (extracted using techniques from music information retrieval), can also be used to measure national life satisfaction and can be more robust than a text-based measure; we also find that the most popular song is superior to the “top 10” or other combinations of high-selling songs. While our methods are specific to one country (the UK) they can be applied to any nation which has readily available music data, which could include anything from recordings to sheet music. The key feature of our work is the ability to convert audio data (as distinct from lyrics) into a simple measure of well-being. This method also has the potential to be applied to periods of the past or to disaggregated groups where no survey data exists.

Our focus for this study is the UK, for which we constructed a Music Valence Index (hereafter MVI) using the valence of the most popular song of each year since the 1970s (according to the official music charts). This valence was predicted by a machine learning model (Support Vector Regression) that had been trained to learn audio features associated with high/low valence according to a separate set of songs that had been annotated by human subjects (Soleymani et al. , 2013). Our methods are described in the Methods section but to provide a simple overview, we first train a machine learning algorithm, composed of 191 different features of sound, to recognize the patterns between audio data and professed well-being in a training set. Our focus is very much on audio data capturing musical attributes (including features such as harmony, timbre, rhythm and melody) and not on lyrics. During training the algorithm is constantly simplified and re-trained until we have a model that accurately measures the relationship between the training set of music samples and the recorded well-being of the listeners. We then apply this trained model to a new set of audio data; in our case, the leading chart music in the UK since the 1970s, which produces a national index of well-being, our MVI. We find that the MVI displays a high and significant degree of similarity with the leading survey-based measure of life satisfaction covering the same period, indicating that audio features embedded within the sound of popular music have the potential to describe national well-being. First, the MVI appears to mirror key aspects in life satisfaction’s variation over time. Second, the two have a significant pairwise correlation, which persists after controlling for national income (GDP), the effect of time and a battery of other controls. Finally, in regression analyses that feature a “horse race” between the MVI and an index that attempts to measure national well-being through text (Hills et al. , 2019), the MVI emerges as a stronger predictor of life satisfaction suggesting that sound may be even more effective than language at teasing out underlying levels of well-being.

The well-being index based on text in Hills et al. (2019) was constructed by analyzing the frequency of a core set of 1000 words in the 8 million books digitized as part of the Google Ngrams corpus for the English language. Each of these words used was allocated a valence score based on subject responses, commonly known as a word norm. Combining word norms with frequency provided a weighted average annual score for valence characterized in Hills et al. (2019) as the national valence index, but renamed the Text Valence Index (TVI) here to reflect the fact that both the TVI and MVI could be considered a national valence index. The TVI provides a means to assess valence alongside survey-data and to provide data from before the availability of survey data, as does the MVI. However, the TVI suffers from the constant evolution of language (which is partially controlled for through removing words that appear to have undergone considerable changes in meaning) and at a practical level requires both a set of word norms and a large digitized language corpus, which are only available for a small number of countries. In contrast the MVI, by considering only audio data (and not lyrics) is naturally robust to changes in the meaning of language over time, and our findings suggest that the most popular single song per year is sufficient to outperform a TVI based upon millions of books.

Many papers have discussed the validity of self-reports of subjective well-being as a measure of national life satisfaction or national happiness, and have concluded that on the whole they are fairly reliable (Diener et al. , 2018). Going beyond survey-based measures and into the realm of natural language processing, as well as Hills et al. (2019), Borowiecki (2019) also conducts a text analysis and links this to well-being, but provides an individual-level analysis, measuring the well-being of three famous composers using the text of their personal letters. To the best of our knowledge, we are the first to use measured emotions in music derived from the audio features contained within sound to make any sort of inference about life satisfaction at the national level.

Our work is supported by a literature on the relationship between music and emotions. The fact that over a hundred studies report that different listeners can hear the same emotions in a song illustrates music’s potential to express emotions (Juslin , 2013). It therefore stands to reason that listeners might choose songs based on their emotional content to help them work through their own emotions. Indeed, previous work shows how music is used to assist with the emotional processing of significant events, to heighten or strengthen the emotional significance of an activity or ritual, and to manage mood (Sloboda and Juslin , 2020). Our results add to this evidence base by showing that the emotions in the most popular songs reflect how people are actually feeling in the population. The psychology of music literature distinguishes between perceived and induced emotions, and it is important to emphasize that the MVI relates only to perceived emotions (the annotated data training the machine learning model concern emotions participants perceived in the music, not how it made them feel); however, this makes it consistent with the notion of music, like a language, being able to describe an emotion to the listener. Whether or not the music has an emotional impact on the listener is therefore not gauged by the MVI (and of course we make no claim that popular music is actually affecting national life satisfaction), but our results (and our success in developing a measure of national life satisfaction) support the idea that the emotions expressed in popular music capture real emotions in the population.

We remain agnostic as to why the measure is successful, but one idea could be that people are more likely to buy a record if it is in tune with how they are feeling, which would imply that the most popular record is then the one that is best able to capture the public mood; this is at least consistent with additional evidence (presented in the Appendix (Table S1)) which demonstrates that the chart-topping song is better able to capture national life satisfaction than tracks further down the charts that are less popular. Note, such a process could be further facilitated by record labels, who would be motivated to promote tracks and artists that tap the public mood if such a strategy is favorable to selling records. Indeed, Hills et al. (2019) suggest a similar mechanism for the TVI in relation to publishing houses and books, and argue that this is strongly suggestive of causation going from national mood to books/newspaper articles (via selection by publishers/editors), rather than the reverse, which might also make sense in the music context. An alternative channel through which national mood could affect the valence of popular music is through the mood of the artists themselves, who might be influenced by the social context. One way to test this with macro data is to see if MVI correlates with led life satisfaction as it is likely there would be some delay for the national mood to filter into artists’ creative output; correlation, however, between MVI and life satisfaction led by one year is insignificant ($r=\text {-}0.196$; $p=0.275$). Nevertheless, national mood affecting musicians’ creative output would still not explain why the top song is better able to capture national mood than songs further down the charts (which can only be rationalized via the demand/listener side of the market).

Our paper also relates to the data science literature on music emotion recognition, a branch of music information retrieval (Kim et al. , 2010). We provide a new application of these methods: correlating the emotions extracted with socio-economic variables.

## Methods

Our methods involve first training a machine learning model to recognize high and low valence in a training set using 191 audio features. This model is then used to construct a Music Valence Index (MVI) based on the predicted valence of the most popular song of the year in the UK from 1973–2010, a time period that enables comparisons with the leading survey-based measure and text-based measure together with a set of controls as detailed below. There is a short description of the method in the introduction; in this section we will look more deeply at the various key stages of the model-building process.

### Training

To predict the valence scores of each song we trained a machine learning model to learn audio features that best predicted valence using a separate set of tracks that had been annotated by human subjects. The annotated dataset comes from Soleymani et al. (2013) (http://cvml.unige.ch/databases/emoMusic/). It consists of 45-s clips of 744 songs from the Free Music Archive (https://freemusicarchive.org/) that span a variety of popular genres (blues, electronic, rock, classical, folk, jazz, country, pop). Each clip was annotated by a minimum of ten participants on a nine-point valence scale, the average of which is our target measure. We considered the average valence a reliable measure for several reasons. First, the dataset was annotated by filtering out most of the participants; only 12.8% of the initial participants succeeded in the qualification test. Second, the authors reported a robust inter-annotation agreement (Krippendorff’s alpha 0.32) for the valence annotations (Soleymani et al. , 2013). In addition, given our application is novel we felt it important to consider the most established methods on music emotion recognition which only use the average valence rather than developing our own.

We computed our own audio features (191 in total) using the 45-s clips (details are provided in the Appendix (Valence Prediction)). Because the valence target exists on an approximately continuous scale (after averaging across participants), we use a regression framework for prediction. Specifically, we use a Support Vector Regression (SVR) which has displayed relatively good performance for predicting valence in comparison to other regression methods (Yang et al. , 2008).

To arrive at our predictive model, we first used a five-fold cross validation procedure to optimize the SVR algorithm’s parameters and the number of features (using $R^2$ to assess performance on the validation sets). We then trained a model using a fraction ($619\approx 83\%$) of the annotated songs and tested its performance on the remaining 125 songs to see how well it might generalize; we were able to achieve a reasonably high $R^2$ on the test set in comparison to machine learning methods from other papers (0.33). Note that we used the same train-test split as in Soleymani et al. (2013) so we could benchmark the model’s performance.

### Application to the UK

We identified the most popular song of the year in the UK using the official singles chart (www.officialcharts.com), which is based on record sales (and includes downloads from 2004 onwards). Only weekly charts are available before 2005 so we applied the following transformation to determine annual scores. Let $x_i$ be a track’s chart position in a given week (1st, 2nd, etc.) and *y* be the lowest possible position on the weekly chart during the year (e.g., 50th, 100th); a track’s popularity score for that year would be calculated as $\sum _{i=1}^{52}(y+1-x_i)$, with the highest-scoring then selected as the most popular. Note, it could be the case that people buy more music during certain weeks of the year (e.g., around Christmas time), so the track we identify as most popular might not have actually obtained the most record sales during the year; rather, the score picks up songs which had lasting popularity over the whole year. The most popular songs were then purchased from Amazon Music or the Apple iTunes Store depending upon availability.

Having collected our core dataset we then re-trained the model on the full sample of 744 annotated songs generating a predicted valence score for the UK’s most popular songs. The song list is available in the Appendix (Table S2), along with each song’s predicted valence. We then extracted 45-s clips from the middle of each song as input data, 22.5 s before and after the middle point of the waveform (to control for song length).

### Validation

To validate the MVI we use Eurobarometer life satisfaction data, specifically the average per year of all individuals surveyed. This is the longest-running measure of subjective well-being (available since 1973), and is also the one used to validate the TVI in Hills et al. (2019). The question asked is, “On the whole, are you very satisfied, fairly satisfied, not very satisfied, or not at all satisfied with the life you lead?”, with responses given on a four-point Likert scale.

The TVI measure from Hills et al. (2019) was constructed using the Google Books corpus (Lin et al. , 2012). They derived annual valence scores for the UK using the average valence of words in books published in Great Britain during a particular year (weighted by their word frequencies). The valence norms used were for 14,000 English words (each an average of valence ratings by 20 participants on a nine-point scale (Warriner et al. , 2013)).

Incorporated in the analyses in the Results section are traditional controls used in the subjective well-being literature. Firstly, our measure of GDP is from the Penn dataset (in 2005 international dollars, adjusted for purchasing power parity). We also use a set of measures from the OECD: life expectancy at birth (as a measure of health); education inequality (measured as a GINI index); total gross central government debt as a percentage of GDP (as a measure of public expenditure); and inflation.

## Results

We will first consider simple scatter plots of the Music Valence Index (MVI) and Text Valence Index (TVI) against life satisfaction before moving on to look at annual changes. These provide a good indication of the strength, direction, and significance of the correlation but it is only when we perform a full regression analysis that we can fully control for important factors such as national income (GDP).

### Time series

**Fig. 1:**
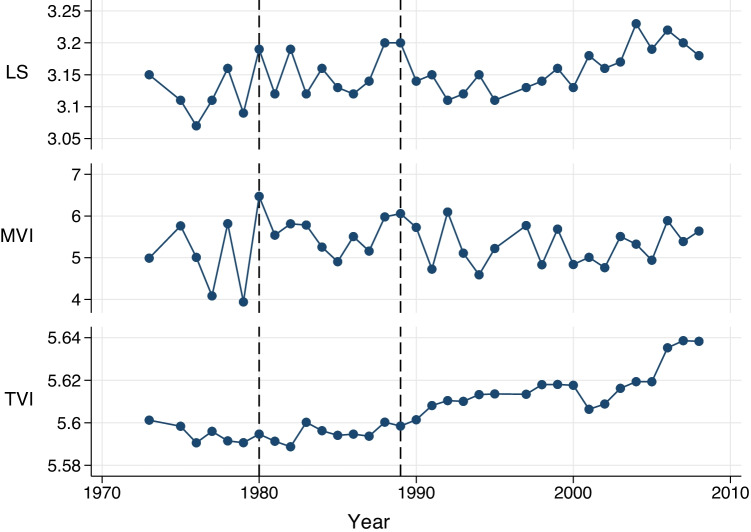
Time series of life satisfaction (LS), the Music Valence Index (MVI) and Text Valence Index (TVI)

As seen in Fig. 1, the MVI displays a high degree of similarity with life satisfaction over time, mirroring key elements in its variation. For example, local peaks in life satisfaction in 1980 and 1989 are picked up by the MVI, which also appears to match well the frequency of the life satisfaction data.

The TVI on the other hand does less well at picking up such peaks, with its frequency resembling that of a smoothed series. This apparent smoothness in the TVI is largely explained by the number of observations involved in the construction of the index. The TVI is composed of millions of books from which the core set of words are extracted, resulting in many millions of observations for each year. This set of observations are then reduced into a single index which represents an average of the valence score for each observation weighted by the frequency of its occurrence: the use of a weighted average over such a large number of observations, of which many are close to invariant to changes in well-being, renders the series very smooth by comparison with the MVI, containing a lower variance and reduced peaks and troughs. Our music index, by comparison, picks up fairly dramatic shifts in emotional content much more easily: in essence the features of our model have been controlled to better detect emotional content and so features that fail to pick up emotion have been removed as part of the machine learning process. This makes our index more capable of picking up peaks and troughs and also partly explains why music seems to correlate well with survey-based well-being which could be considered to be the underlying ground-truth.

### Scatter plots

**Fig. 2:**
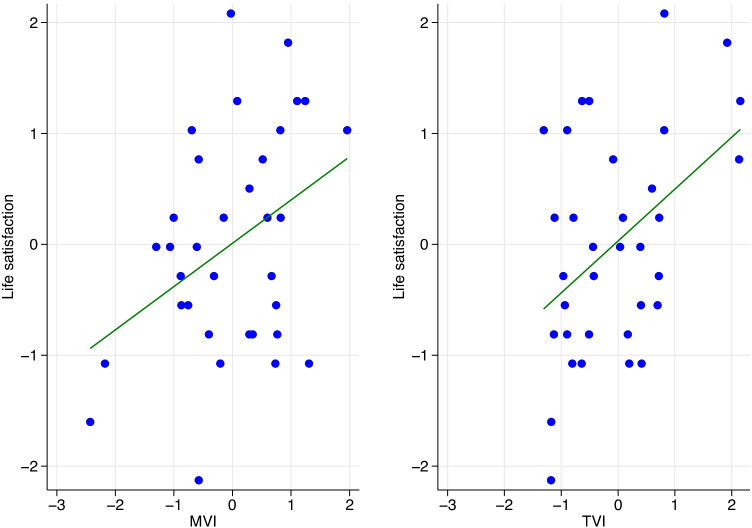
Scatter plot of life satisfaction and the Music Valence Index (MVI) alongside Life Satisfaction and the Text Valence Index (TVI)

Figure 2 shows a scatter plot of life satisfaction and the MVI alongside a similar scatter plot for life satisfaction and the TVI. In order to facilitate a visual comparison, all variables in Fig. 2 have been standardized (subtracting the mean and dividing by the standard deviation), and the same scale used for the axes. The relationship between life satisfaction and both indices is positive and significant (for MVI: $r=0.38$; $p=0.02$, for TVI: $r=0.45$; $p=0.01$). While in the simple scatter plot we see a slightly stronger correlation between TVI and life satisfaction, this does not take into account important factors such as GDP and the unobserved effects of time which become apparent in both the regression analysis and to some extent the next pair of figures.

### Annual changes

**Fig. 3:**
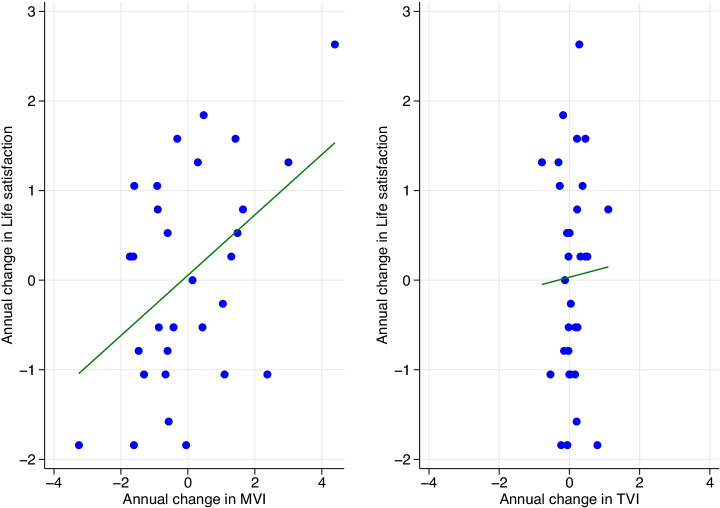
Scatter plot of annual change in life satisfaction and annual change in the Music Valence Index (MVI) alongside annual change in life satisfaction and annual change in the Text Valence Index (TVI)

We next consider the annual change in the MVI or TVI as compared with the annual change in life satisfaction in Fig. 3.

In Fig. 3, once again all variables have been standardized (before being differenced), and the same scales are used on the axes. We can see that there is a much stronger correlation of change in MVI on change in life satisfaction ($r=0.46$; $p<0.01$) than change in TVI on change in life satisfaction ($r=0.03$; $p=0.86$). This suggests how confounding factors (such as unobserved (linear) effects of time) have contributed to the strong raw correlation between TVI and life satisfaction (in levels), as when such effects are removed through differencing, TVI’s correlation significantly weakens. Of course we still do not control for a number of factors in these scatter plots which is something we will address in the regression analysis to follow.

### Regression analysis

Regression analyses in Table 1 (specifications (1) and (2)) show that the positive relationship between MVI and life satisfaction is robust to the introduction of GDP, a linear time trend and various other controls ($p=0.003$ without the additional controls; $p=0.008$ with them). In all regression analyses we report heteroskedasticity-consistent (or Eicker–Huber–White) standard errors, but there are no substantive differences in the results with classical standard errors.

**Table 1:** The Music Valence Index (MVI) predicts life satisfaction

Marginal effects	Life satisfaction			
	(1)	(2)	(3)	(4)
MVI	0.403$$^{***}$$	0.400$$^{***}$$	0.406$$^{***}$$	0.417$$^{***}$$
	(0.126)	(0.139)	(0.129)	(0.143)
TVI			${-}$0.101	${-}$0.277
			(0.237)	(0.347)
GDP	6.632$^{*}$	6.832	6.665$^{*}$	6.657
	(3.834)	(4.702)	(3.867)	(4.645)
Trend	Yes	Yes	Yes	Yes
Other controls	No	Yes	No	Yes
Observations	34	34	34	34

$$^{***} p<0.01$$; $$^{**} p<0.05$$; $$^* p<0.1$$. Marginal effects with (heteroskedasticity-consistent) standard errors in parentheses. Life satisfaction, MVI and TVI are standardized; GDP is the logarithm of gross domestic product per capita. Other controls include life expectancy, education inequality, public debt and inflation

Next we consider the relative strength of our MVI as compared with a text-based measure when the two are pitted against each other. To do so, we perform a regression analysis with both of our candidate predictors, the MVI and TVI, situated on the right-hand side of the regression, which is commonly referred to in the literature as a “horse race”. Rather than attempting to suggest that either variable has a causal effect on life satisfaction (the more common use of a regression), this technique instead seeks to evaluate which is a stronger predictor, or alternatively which has a stronger correlation, measurable using *p* value. As shown in specifications (3) and (4) of Table 1, when included in the same regression, the MVI emerges as a stronger predictor of life satisfaction than the TVI for the UK, with a coefficient that remains significant. This holds true whether the full set of controls (life expectancy, education inequality, public debt, and inflation) are included or not ($p=0.004$ without the additional controls; $p=0.007$ with them).

## Conclusion

In this paper we have provided evidence that the valence of a country’s most popular songs can provide a reliable indication of average life satisfaction in the population. This might be considered surprising: not everyone listens to music and indeed listening to “chart-topping” music might even be considered largely a teenage pass-time. However, it is clear from our results that the audio features embedded within the sound of chart-topping music do correlate well with national well-being. This could be because the most popular chart hit in any given year goes beyond the traditional pop music demographic and is more representative of national mood, it could be because those who buy popular music do in fact provide a reasonable sample of the population, or it might provide a reasonable proxy for some other reason. What is clear is that for whatever reason the correlation between the Music Valence Index (MVI) and national well-being as measured through more traditional survey-based measures is strong and highly significant.

Moreover, for the UK at least, it appears that the valence of popular music provides a more accurate depiction of national life satisfaction than the valence enshrined within books, which provides even greater support for the idea of music as a specialized “language of the emotions” (Cooke , 1959). A nice feature of our measure is that it only requires collecting information on one song each year (the most popular), which makes it relatively cheap and easy to implement. We support this further in the Appendix (Table S1) where we show that using the valences of tracks that are less popular (including an average of the top 10 songs) does not work as well as focusing only on chart-topping songs. It might also be interesting to note that the pairwise correlation between the MVI and life satisfaction falls to only 0.15 (and becomes insignificant) when we consider life satisfaction lagged by one year. This is in stark contrast to the Text Valence Index (TVI) which improves when we lag life satisfaction. This suggests that music is also a more immediate measure of national mood which could hint at the efficiency of the music industry, or alternatively suggest that popular music is more ephemeral than literature.

Here we have shown that music can predict life satisfaction within a country. Future research might wish to consider the potential for music to explain between-country differences in life satisfaction. Music has the potential to be a good between-country predictor since it is not only an emotional language but a “universal” one (Longfellow , 1835) and is found in every society with a stable set of functions (Mehr et al. , 2019). Data availability is improving over time: for the UK downloads were incorporated in music chart data in 2004, streaming was partially added from 2008 and fully incorporated from 2014 onwards. With downloads and streaming becoming increasingly prevalent it will be easier to measure listening behaviour accurately. There is also scope for examining both the role of different genres of music (as they compete for an audience) and the changes in valence within genres (which might link to the mood of specific groups who are more likely to listen to these genres) building on existing work on the evolution of music (Mauch et al. , 2015). We also note here that the TVI stops in 2010 which is the point at which Google’s digitization of books ended when Hills et al. (2019) was produced. Google has and continues to update the digitization of books. In the future it would be interesting to update both the TVI and MVI and continue assessing the extent to which they correlate well with different survey-based measures of well-being, as well as to explore the impact of important shocks to well-being that have occurred since 2010 such as major political shocks, the rise of populism, Brexit in the UK, the COVID-19 pandemic, ongoing climate change and many other important and ongoing events. As more data accumulates it might also be interesting to explore the impact of downloads and streaming on the consistency of the well-being measure. Finally, we might also wonder about the differential impact on well-being of domestic vs foreign music. We see some evidence that music from non-UK artists generates a positive and significant correlation between the MVI and life satisfaction as we might expect (r=0.504; p=0.017), but we do not have enough observations to be sure of the relationship when we restrict our sample to UK artists (negative but highly insignificant, r =-0.020; p=0.951). In short, across all of these areas, our work should be seen as introducing a new way to measure well-being that can and hopefully will be continuously updated in the future.

Our focus has been on the musical characteristics of audio data (such as melody, harmony, timbre and rhythm) which provides a very distinct break from text-based attempts to study mood. This means that we did not consider lyrics. This has a number of advantages particularly when we consider the generality of the method. Many cultures have primarily instrumental music which feature no lyrics. Others may incorporate music from foreign cultures and so we cannot know the extent to which lyrics matter. Audio data is naturally robust to these considerations which may explain the notion of music having the potential to be universal.

In general, we hope to encourage a closer look at the emotions contained within music and the greater use of audio data as potentially representative of underlying social and cultural patterns. We also want to emphasize the ability to use audio data to investigate well-being in the past and for disaggregate subgroups where survey data is not available.

## Supplementary Information

ESM 1 (PDF 71 kb)
