# Supplementary material for: Measuring national mood with music: using machine learning to construct a measure of national valence from audio data
Source: Behav Res Methods. 2022 Feb 25;54(6):3085–92. doi: 10.3758/s13428-021-01747-7 (PMC8876081; doi:10.3758/s13428-021-01747-7)
Supplement: Supplementary file 1 — (PDF 71 kb) [file 13428_2021_1747_MOESM1_ESM.pdf]

Appendix

This Appendix provides some additional detail on the underlying methodology which might be of interest to specialists in the area or those interested in replicating our findings. We also provide direct evidence that the most popular song is the best predictor of life satisfaction. Finally, we provide the valence levels of the individual songs used.

Valence Prediction

We extracted commonly used acoustic features for music emotion recognition (Kim et al., 2010) using the music processing libraries Librosa (McFee et al., 2015) and Essentia (Bogdanov et al., 2013): Spectral Centroid; Spectral Rolloff; Spectral Contrast - 7 bands; Mel-Frequency Cepstrum Coefficients (MFCC) - 24 coefficients; Zero Crossing Rate; Chroma Energy Normalized Statistics (CENS) - 12 chroma; Beat Per Minute (BPM); Root Mean Square (RMS); Spectral Flux; Onset Rate; and High Frequency Content (HFC).

For frame-level features, we used Hann windows of 46 ms, and computed the mean and variance of the frame values and first-order differences. For spectral flux and HFC we computed only the mean and the variance of frame values. In total there were 191 features.

We then trained a Support Vector Regressor (SVR) on the annotated Free Music Archive dataset using radial basis functions as kernels. Features were preprocessed with z-score normalisation (removing the mean and scaling to unit variance) so features with large magnitude would not dominate the objective function. A 5-fold cross-validation procedure selected the optimal parameters of the SVR algorithm and number of features (100). Feature selection was carried out using the F-test which tests the individual effect of each feature by converting the correlation between each feature and the valence to an F score. Using the same train-test split as in Soleymani et al. (2013), our achieved R^2 on the test set compares favourably with other machine learning models as indicated in the following table:

Method	Valence R^2
This Paper	0.33
Baseline[a]	0.12
MFCC[b]	0.20
TUM[c]	0.42
UAizu[c]	0.35
UU[c]	0.31

[a]Soleymani et al. (2013), [b]Choi et al. (2017), [c]Soleymani et al. (2014)

Table S1: The Most Popular Song is the Best Measure of Life Satisfaction

Correlations *(p)*	Life Satisfaction
**Valence of #1 Song (MVI)**	0.385** (0.025)
Valence of #2 Song	0.128 (0.470)
**Valence of #3 Song**	0.314* (0.070)
**Valence of #4 Song**	0.344* (0.054)
Valence of #5 Song	-0.161 (0.364)
Valence of #6 Song	0.026 (0.885)
Valence of #7 Song	0.017 (0.924)
Valence of #8 Song	-0.157 (0.375)
Valence of #9 Song	0.249 (0.155)
Valence of #10 Song	0.017 (0.924)
**Average Valence of #1-#10 Songs**	0.311* (0.073)

Pairwise correlations with p-values in parentheses. Statistically significant measures presented in bold: $^{**}p < 0.05$; $^{*}p < 0.1$.

Table S2: Most Popular Songs of the Year and their Predicted Valences (which form the MVI)

Year	Title	Artist	Valence *(1-9)*
1973	Tie a Yellow Ribbon Round the Ole Oak Tree	Dawn featuring Tony Orlando	4.99
1974	The Wombling Song	The Wombles	5.40
1975	Bye Bye Baby	Bay City Rollers	5.76
1976	Mississippi	Pussycat	5.01
1977	Evergreen	Barbra Streisand	4.08
1978	Rivers of Babylon	Boney M.	5.82
1979	Bright Eyes	Art Garfunkel	3.94
1980	Feels Like I'm in Love	Kelly Marie	6.47
1981	Birdie Song	The Tweets	5.54
1982	Come On Eileen	Dexy's Midnight Runners	5.81
1983	Blue Monday	New Order	5.78
1984	Relax	Frankie Goes To Hollywood	5.25
1985	The Power of Love	Jennifer Rush	4.90
1986	So Macho	Sinitta	5.51
1987	Never Gonna Give You Up	Rick Astley	5.16
1988	Push It	Salt-N-Pepa	5.98
1989	Ride on Time	Black Box	6.06
1990	Killer	Adamski	5.73
1991	(Everything I Do) I Do It for You	Bryan Adams	4.73
1992	Rhythm Is a Dancer	Snap!	6.10
1993	No Limit	2 Unlimited	5.11
1994	Love Is All Around	Wet Wet Wet	4.59
1995	Think Twice	Celine Dion	5.22
1996	Return of the Mack	Mark Morrison	5.98
1997	I'll Be Missing You	Puff Daddy & Faith Evans	5.77
1998	How Do I Live	LeAnn Rimes	4.83
1999	Heartbeat	Steps	5.69
2000	Amazed	Lonestar	4.84
2001	Whole Again	Atomic Kitten	5.01
2002	How You Remind Me	Nickelback	4.76
2003	In Da Club	50 Cent	5.51
2004	Left Outside Alone	Anastacia	5.33
2005	You're Beautiful	James Blunt	4.94
2006	Hips Don't Lie	Shakira featuring Wyclef Jean	5.89
2007	How to Save a Life	The Fray	5.39
2008	Rockstar	Nickelback	5.64
2009	Poker Face	Lady Gaga	6.01
2010	Empire State of Mind	Alicia Keys	4.45
